# Long-term cardiovascular outcomes of gestational diabetes mellitus: a prospective UK Biobank study

**DOI:** 10.1186/s12933-022-01663-w

**Published:** 2022-10-29

**Authors:** Seung Mi Lee, Manu Shivakumar, Ji Won Park, Young Mi Jung, Eun Kyung Choe, Soo Heon Kwak, Sohee Oh, Joong Shin Park, Jong Kwan Jun, Dokyoon Kim, Jae-Seung Yun

**Affiliations:** 1 Department of Obstetrics and Gynecology, Seoul National University Hospital, Seoul, Korea; 2 Department of Obstetrics and Gynecology, Seoul National University College of Medicine, Seoul, Korea; 3 Department of Biostatistics, Epidemiology & Informatics, Perelman School of Medicine, University of Pennsylvania, B304 Richards Building, 3700 Hamilton Walk, Philadelphia, PA 19104-6116 USA; 4 Department of Surgery, Seoul National University College of Medicine, Seoul, Korea; 5 Department of Surgery, Seoul National University Hospital Healthcare System Gangnam Center, Seoul, Korea; 6 Department of Internal Medicine, Seoul National University College of Medicine, Seoul, Korea; 7 Transdisciplinary Department of Medicine & Advanced Technology, Seoul National University Hospital, Seoul, Korea; 8 Department of Biostatistics, Seoul Metropolitan Government Seoul National University Boramae Medical Center, Seoul, Korea; 9 Department of Internal Medicine, Catholic University College of Medicine, 222, Banpo-Daero, Seocho-Gu, Seoul, Republic of Korea

**Keywords:** Gestational diabetes mellitus, Long-term outcomes, Cardiovascular outcome, UK Biobank

## Abstract

**Background:**

Previous studies showed that gestational diabetes mellitus (GDM) can be a risk factor for subsequent atherosclerotic cardiovascular disease. However, there is a paucity of information regarding diverse cardiovascular outcomes in elderly women after GDM. In the current study, we examined whether women with a history of GDM have an increased risk for long-term overall cardiovascular outcomes.

**Methods:**

Among the UK participants, we included 219,330 women aged 40 to 69 years who reported at least one live birth. The new incidence of diverse cardiovascular outcomes was compared according to GDM history by multivariable Cox proportional hazard models. In addition, causal mediation analysis was performed to examine the contribution of well-known risk factors to observed risk.

**Results:**

After enrollment, 13,094 women (6.0%) developed new overall cardiovascular outcomes. Women with GDM history had an increased risk for overall cardiovascular outcomes [adjusted HR (aHR) 1.36 (95% CI 1.18–1.55)], including coronary artery disease [aHR 1.31 (1.08–1.59)], myocardial infarction [aHR 1.65 (1.27–2.15)], ischemic stroke [aHR 1.68 (1.18–2.39)], peripheral artery disease [aHR 1.69 (1.14–2.51)], heart failure [aHR 1.41 (1.06–1.87)], mitral regurgitation [aHR 2.25 (1.51–3.34)], and atrial fibrillation/flutter [aHR 1.47 (1.18–1.84)], after adjustment for age, race, BMI, smoking, early menopause, hysterectomy, prevalent disease, and medication. In mediation analysis, overt diabetes explained 23%, hypertension explained 11%, and dyslipidemia explained 10% of the association between GDM and overall cardiovascular outcome.

**Conclusions:**

GDM was associated with more diverse cardiovascular outcomes than previously considered, and conventional risk factors such as diabetes, hypertension, and dyslipidemia partially contributed to this relationship.

**Supplementary Information:**

The online version contains supplementary material available at 10.1186/s12933-022-01663-w.

## Introduction

Cardiovascular disease (CVD) is the leading cause of mortality worldwide, accounting for the death of one in every three women [1, 2]. Identification of high-risk subjects and application of active prevention is essential, and there is increasing recognition that sex-specific factors should be incorporated in risk assessment. Among women-specific features, there is accumulating evidence that pregnancy complications are important risk factors for future cardiovascular disease [3–8]. GDM, defined as glucose intolerance first diagnosed during pregnancy, is one of the major complications during pregnancy. Various mechanisms have been suggested for impaired glucose tolerance during pregnancy and GDM is associated with short- and long-term fetal and childhood health, such as adulthood carbohydrate metabolism disturbances [9–12]. For maternal health, previous studies suggested that a history of GDM can be a risk factor for subsequent maternal diabetes, hypertension [13], dyslipidemia [14], and atherosclerotic cardiovascular diseases (ASCVDs) such as myocardial infarction or ischemic stroke [5, 15, 16].

However, key limitations of previous studies need to be addressed. First, most studies followed up subjects for a relatively short-term period with a follow-up period of 10–20 years after pregnancy [4–6, 17–23]. Until now, studies on the long-term outcome of GDM in the elderly population have been lacking, and few studies have reported the following outcomes in women aged 60–70 years. Second, previous studies mainly focused on ASCVD, hence the effect of GDM on non-atherosclerotic cardiovascular diseases (non-ASCVD) remains unknown [4–6, 17–23]. Third, few studies have issued the impact of chronic metabolic comorbidities on the development of a cardiovascular outcome. As chronic metabolic diseases such as diabetes, hypertension, and dyslipidemia are known to occur more frequently in women with a history of GDM and these morbidities are strong risk factors for cardiovascular outcomes, the mediation effect of these chronic metabolic diseases should also be considered in the evaluation of cardiovascular risk after GDM [24].

The UK Biobank is a prospective cohort study that recruited participants 40 to 69 years old with ongoing follow-up [25, 26]. During the follow-up of health outcomes, the UK biobank collected data from various sources, including primary care, national hospital inpatient and outpatient records, enabling the capture of various health outcomes. Because of these points, data from the UK Biobank can be used to evaluate (1) the long-term outcome of GDM in the elderly population; (2) diverse cardiovascular outcomes including not only atherosclerotic heart diseases but also non-atherosclerotic heart diseases; and (3) the mediating effect of chronic morbidities on cardiovascular risk after GDM.

The purpose of the current study was to examine whether women with a history of GDM have an increased risk for long-term various cardiovascular outcomes using data from the UK Biobank.

## Methods

### Data source

The UK Biobank is a population-based prospective cohort study that recruited > 500,000 adult residents aged 40 to 69 years at enrollment between 2006 and 2010 with ongoing follow-up [25, 26]. At the time of enrollment, participants gave written informed consent, provided information by questionnaire regarding demographic data, lifestyle, environmental and medical history, and had physical measurements taken. For ongoing follow-up, the UK Biobank has been collecting incident disease diagnoses gleaned from linkage of various datasets including primary care, national hospital inpatient and outpatient records, and death registrations [25].

This study was covered by ethical approval for studies using the UK Biobank from the Northwest Multi-center Research Ethics Committee (MREC) (June 17, 2011 [reference 11/NW/0382]; extended on May 13, 2016 [reference 16/NW/0274]).

### Study design

Among women enrolled at 40–69 years, we included women who reported at least one live birth. For the current analysis, we excluded women who were diagnosed with overt diabetes before the index pregnancy that accompanied GDM. Additionally, women who did not have a history of GDM before enrollment but were subsequently diagnosed with GDM after enrollment were also excluded. For the possible association between congenital heart disease and cardiovascular outcomes, women with congenital heart disease were excluded, according to the International Classification of Diseases (ICD) codes (Additional file 1: Table S1).

GDM was defined by self-report from participants at enrollment or by the ICD codes. At enrollment, all the female participants were asked whether they had a history of GDM during pregnancy either by a verbal interview or touchscreen questionnaire. Additionally, the UK Biobank also collected data regarding disease diagnoses that were recorded before enrollment, and GDM was defined using ICD codes (Additional file 1: Table S1). To exclude women with overt diabetes before pregnancy, we excluded those whose diagnosis for diabetes mellitus was made before the diagnosis of GDM. Prevalent comorbidities at enrollment were captured either from self-report at enrollment or disease diagnosis before enrollment using ICD codes.

The primary outcome was designated as a composite of a new occurrence of cardiovascular outcomes, including coronary artery disease, myocardial infarction, ischemic stroke, peripheral artery disease, heart failure, aortic stenosis, mitral regurgitation, atrial fibrillation/flutter, and venous thromboembolism. The secondary outcome was constituted by each cardiovascular outcome included in the primary outcome. A composite of atherosclerotic cardiovascular disease (ASCVD, a composite of coronary artery disease, myocardial infarction, ischemic stroke, and peripheral artery disease) and a composite of non-ASCVD (a composite of heart failure, aortic stenosis, mitral regurgitation, atrial fibrillation/flutter, and venous thromboembolism) was also evaluated. ICD codes for each outcome are described in Additional file 1: Table S1. In addition, myocardial infarction and ischemic stroke were algorithmically defined by the UK Biobank [27].

New occurrence of cardiovascular outcomes was defined as CVD events that occurred after the enrollment date. If the diagnosis of a specific CVD outcome was made several times, the occurrence of that event was censored for the first date of diagnosis to exclude multiple events. For the occurrence of composite outcome such as total cardiovascular outcome, ASCVD, and non-ASCVD, events were censored at first CVD event, whichever came first. For subtypes of cardiovascular outcomes, the occurrence of that event was censored for the first date of diagnosis of each subtype of CVD outcome.

### Statistical analysis

Baseline characteristics of UK Biobank participants with and without GDM were compared using Student’s *t*-test or Mann–Whitney U test for continuous variables and Pearson chi-square test or Fisher’s exact test for categorical variables as appropriate. In the analyses for subtypes of CVD, participants with each CVD history were excluded per subtype of CVD outcome. For example, women with prevalent coronary artery disease at enrollment were excluded from the models for coronary artery disease.

To evaluate the new occurrence of cardiovascular outcomes, a Cox proportional hazard model was used to calculate hazard ratios with 95% confidence intervals after adjustment for covariates including age, race, BMI, smoking, alcohol consumption, early menopause, hysterectomy, prevalent diseases (hypertension, diabetes, or dyslipidemia), and medication (aspirin, antihypertensive, cholesterol-lowering agent). At enrollment, the UK Biobank retrieved various characteristics including lifestyle factors such as smoking and sex-specific variables including menopause or hysterectomy. Prevalent diseases were captured either from self-report at enrollment or disease diagnosis before enrollment using ICD codes. Medication was also captured from self-report. Time-to-censoring was started from the date of enrollment and ended by the date of new occurrence of each disease for cases or by death or last follow-up for non-cases.

To evaluate the effect of GDM after reduction of the bias from covariates, subgroup analysis after propensity score matching was also performed. The control group was selected after matching for age, race, BMI, smoking, alcohol consumption, early menopause, hysterectomy, prevalent diseases (hypertension, diabetes, or dyslipidemia), and medication (aspirin, antihypertensive, cholesterol-lowering agent) at a ratio of 1:5 and nearest-neighbor matching.

In addition, we performed causal mediation analysis to examine the contribution of well-known risk factors to observed cardiovascular risk. For this, the contribution of prevalent diabetes, hypertension, and dyslipidemia to the risk of the new occurrence of cardiovascular outcomes associated with GDM was analyzed after adjustment for other conventional cardiovascular risk factors (age, race, BMI at enrollment, ever smoking, alcohol consumption, in addition to subsequent diabetes, hypertension, and dyslipidemia). Each mediation analysis was performed with 1,000 simulations with a quasi-Bayesian method to estimate variance. A *p* value less than 0.05 was considered statistically significant. R version 4.0.3 was used for the analysis.

## Results

### Subject population

**Fig. 1:**
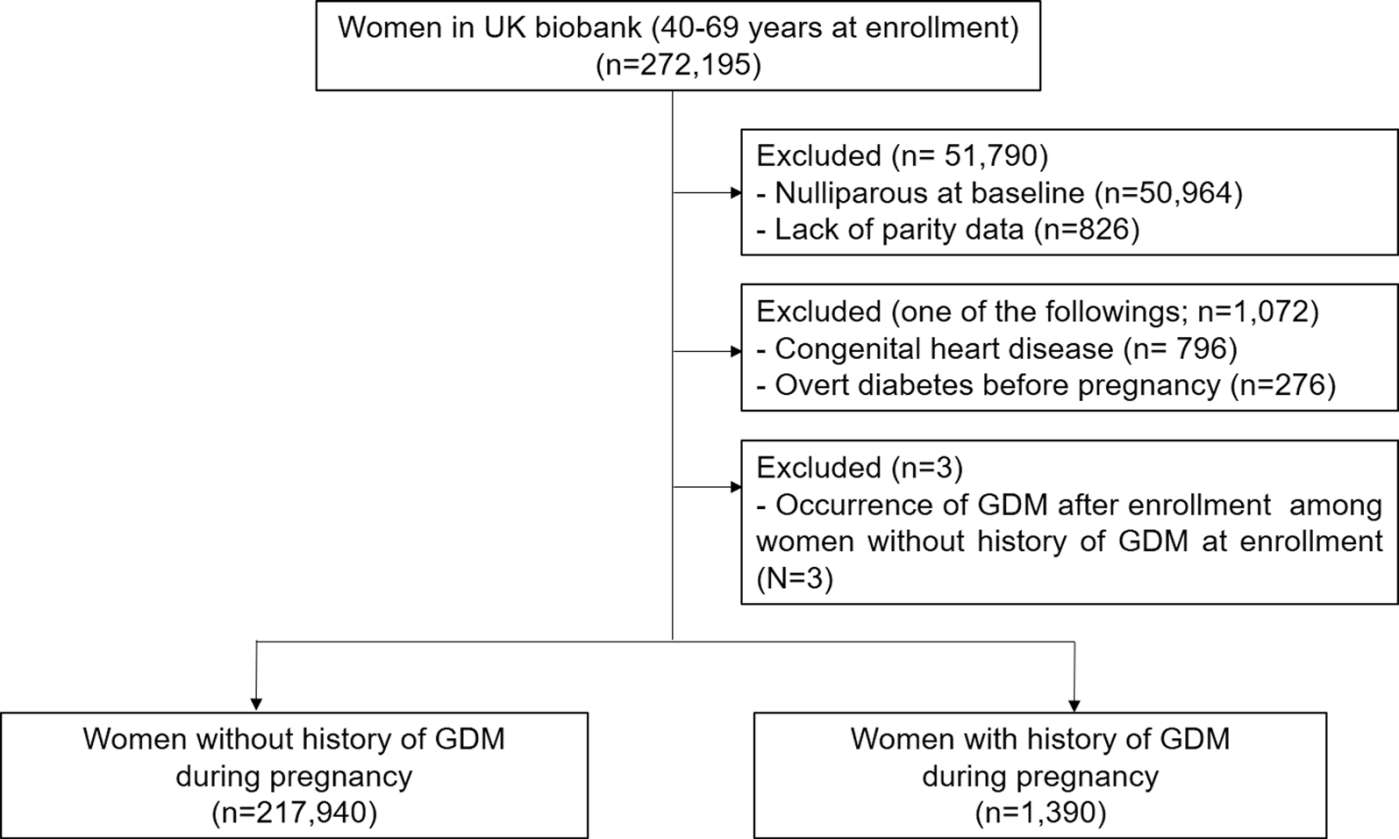
Study population *GDM* gestational diabetes mellitus

Among 272,195 women enrolled at 40–69 years, a total of 219,330 women met the inclusion criteria and were retained for the analysis (Fig. 1). Among them, 1,390 women were diagnosed with GDM during pregnancy.

Table 1 shows the baseline characteristics of the study population. Women with a history of GDM had a lower age at enrollment and were less likely to be Caucasian. In reproductive history, women with a history of GDM delivered their first baby at an older age. At enrollment, women with a history of GDM had a higher BMI, and the frequency of ever smoking was lower in these women.

**Table 1:** Baseline clinical features and prevalent diseases of the study population

Characteristics	No history of GDM	History of GDM
	(n = 217,940)	(n = 1,390)
Age (years)	56.9 ± 7.8	52.1 ± 8.1
Caucasian	205,724 (94.4)	1,151 (82.8)
BMI (kg/m^2^)	27.1 ± 5.1	29.2 ± 6.0
Obesity (BMI > 30 kg/m^2^)	51,487 (23.7)	521 (37.8)
Age at first live birth (years)	25.3 ± 4.6	26.5 ± 5.4
Mean duration between first birth and enrollment (years)	31.9 ± 9.7	25.9 ± 10.8
Early menopause < 40 years (years)	5,498 (2.5)	46 (3.3)
Hysterectomy	18,460 (8.5)	91 (6.5)
Ever smoking	88,563 (40.6)	500 (36.0)
Prevalent comorbidity at baseline		
Type 2 diabetes	7,769 (3.6)	414 (29.8)
Hypertension	55,825 (25.6)	501 (36.0)
Dyslipidemia	26,249 (12.0)	301 (21.7)
Use of medication		
Aspirin	22,188 (10.2)	234 (16.8)
Anti-hypertensive agent	41,595 (19.1)	387 (27.8)
Lipid-lowering agent	30,777 (14.1)	395 (28.4)

Data are presented as proportion (%) or mean ± standard deviation

*BMI* body mass index, *GDM* gestational diabetes mellitus

### Prevalent diseases at enrollment

**Fig. 2:**
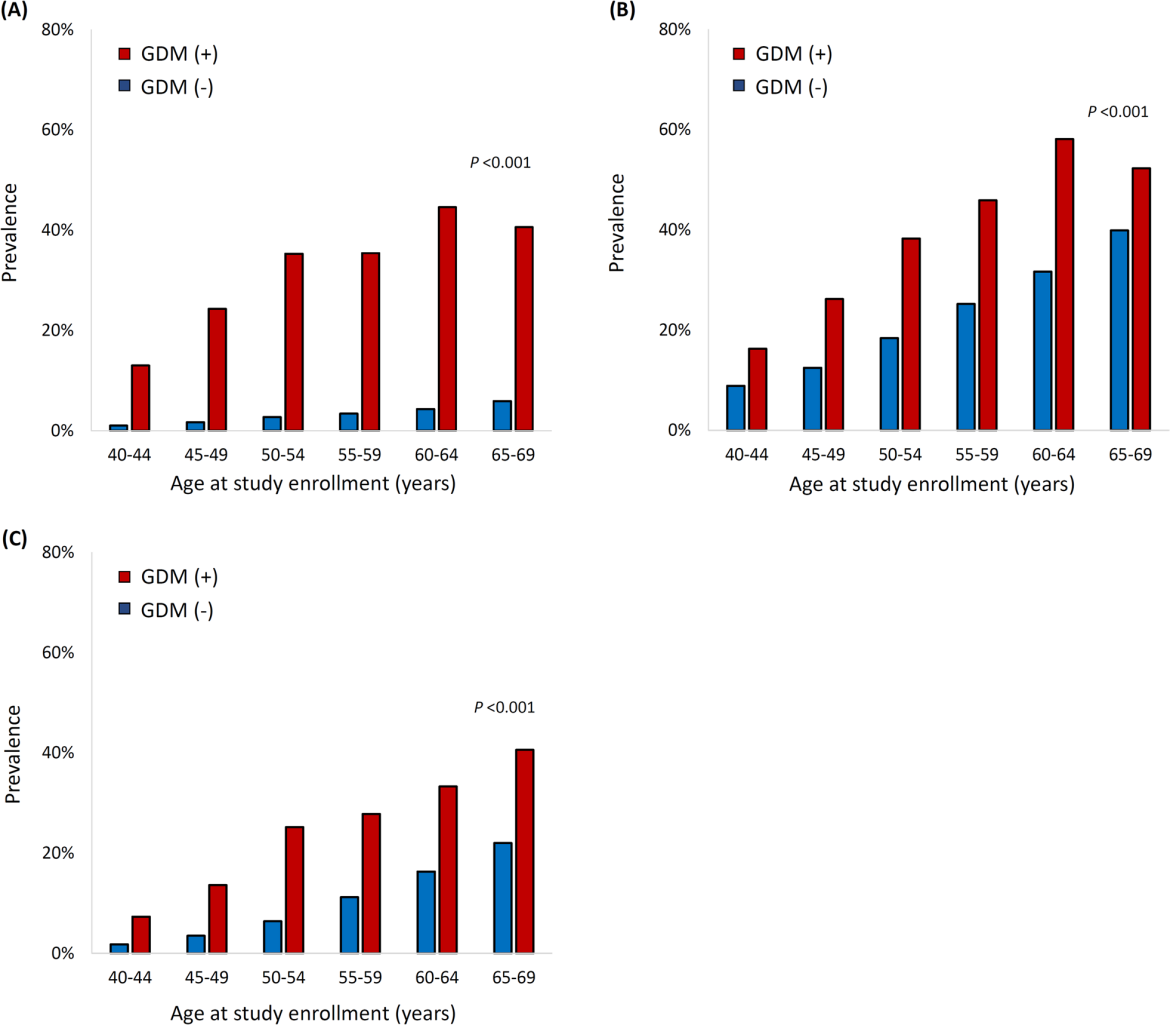
Frequency of prevalent diabetes, hypertension, and dyslipidemia by the age at enrollment (**a**) Diabetes (**b**) Hypertension (**c**) Dyslipidemia

For prevalent diseases, women with a history of GDM had a higher frequency of comorbidities such as diabetes, hypertension, and dyslipidemia and were more likely to have medications such as aspirin, antihypertensive agents, and cholesterol-lowering agents. Specifically, the frequency of prevalent diabetes was much higher in women with a history of GDM than in those without, in each age group, and the frequency of prevalent hypertension and dyslipidemia also showed a similar pattern (Fig. 2).

In terms of cardiovascular outcomes, the frequency of prevalent cardiovascular diseases was also increased at enrollment for some cardiovascular diseases, such as coronary artery disease, myocardial infarction, and heart failure (Additional file 1: Table S2).

### Risk of new occurrence of cardiovascular outcomes

**Fig. 3:**
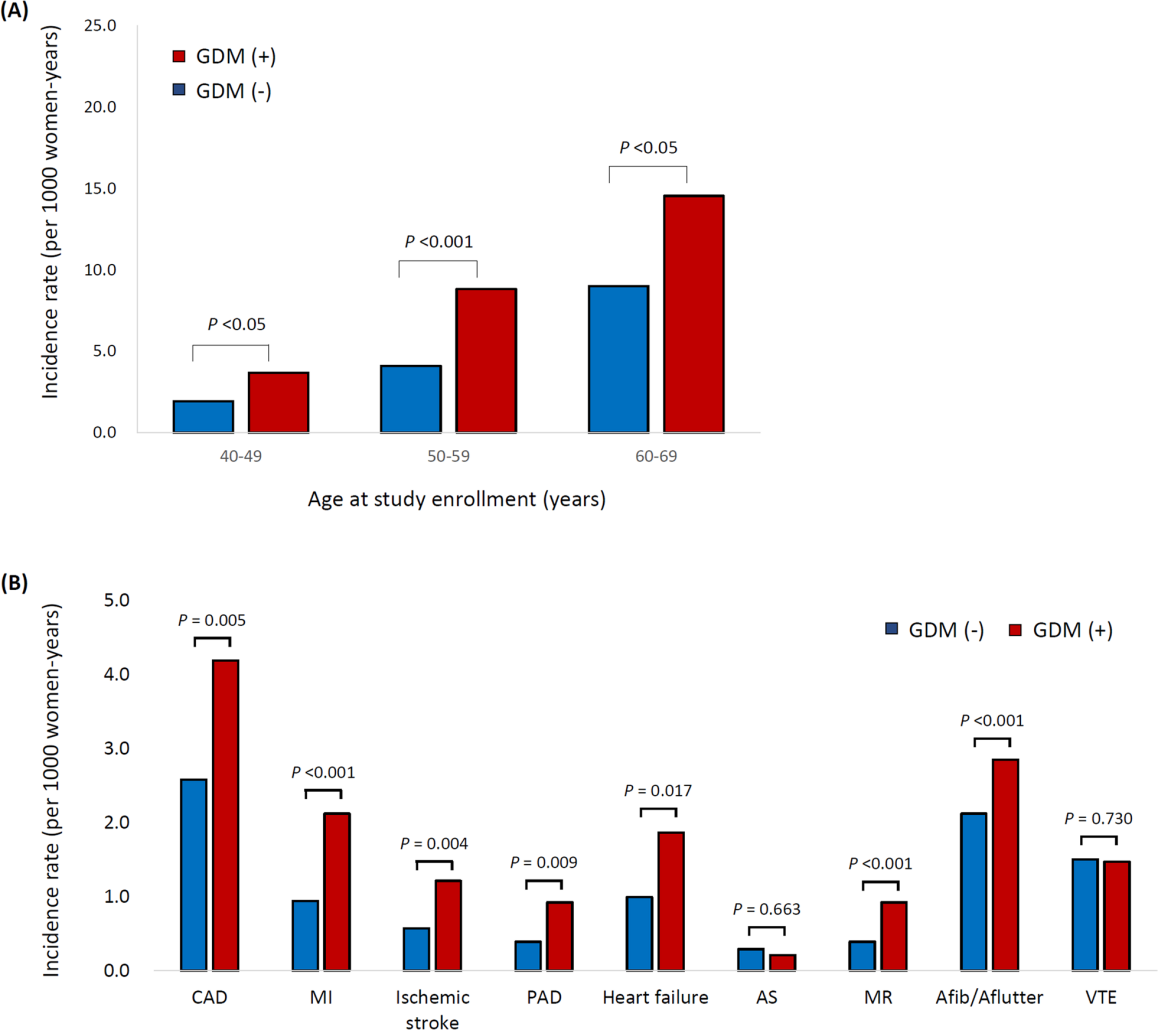
Occurrence of incident cardiovascular outcomes during follow up (**a**) Incidence of total cardiovascular outcome by the age at enrollment (**b**) Incidence of each cardiovascular outcomes p value: Adjusted for age at enrollment, race, BMI, smoking, alcohol consumption, early menopause, hysterectomy, prevalent comorbidities (hypertension, diabetes, dyslipidemia) and medication by Cox regression analysis *CAD* coronary artery disease, *MI* myocardial infarction, *PAD* peripheral artery disease, *AS* aortic stenosis, *MR* mitral regurgitation, *Afib* atrial fibrillation, *Aflutter* atrial flutter, *VTE* venous thromboembolism

After enrollment, the median follow-up duration was 10.3 years, and a total of 13,094 women were newly diagnosed with cardiovascular diseases. The risk of a new occurrence of the primary outcome (total cardiovascular outcomes) was increased in women with a history of GDM. The incidence of cardiovascular outcome was significantly higher in women with a history of GDM than in those without prior GDM (p = 0.008 for all ages), and this increased risk of total cardiovascular outcome was observed in each age group Fig. 3(a, b) compares the number of total cardiovascular outcomes per 1,000 women-year of follow-up. This increased risk was noted in most cardiovascular diseases, including coronary artery disease, myocardial infarction, ischemic stroke, peripheral artery disease, heart failure, mitral regurgitation, and atrial fibrillation.

**Fig. 4:**
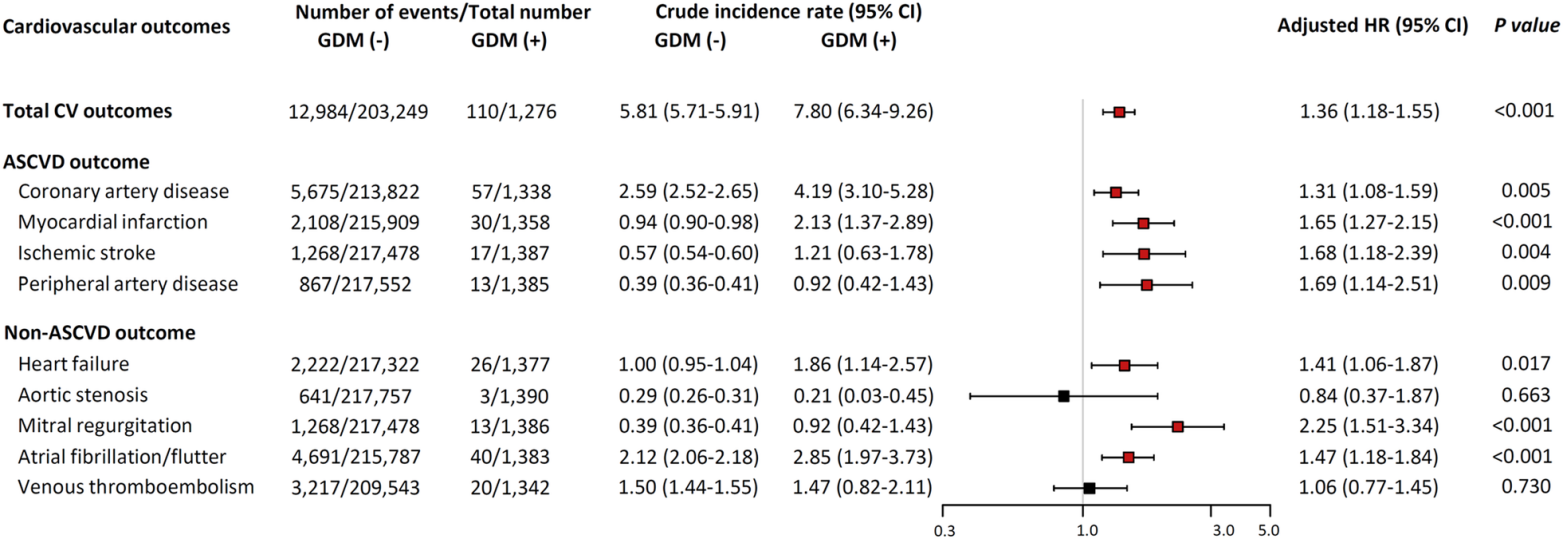
Hazard ratio of each cardiovascular outcomes

Table 2 and Fig. 4 show the number of occurrences and the hazard ratio of each cardiovascular outcome after adjustment for age, race, BMI, smoking, early menopause, hysterectomy, prevalent diseases (hypertension, diabetes, or dyslipidemia), and medication (aspirin, antihypertensive, cholesterol-lowering agent) by Cox proportional hazards regression analysis. Specifically, women with a history of GDM had an increased risk for the new occurrence of total cardiovascular outcomes (HR 1.36, p < 0.001), coronary artery disease (HR 1.31, p = 0.005), myocardial infarction (HR 1.65, p < 0.001), ischemic stroke (HR 1.68, p = 0.004), peripheral artery disease (HR 1.69, p = 0.009), heart failure (HR 1.41, p = 0.017), mitral regurgitation (HR 2.25, p < 0.001), and atrial fibrillation/flutter (HR 1.47, p < 0.001).

**Table 2:** Incident diagnosis of cardiovascular outcomes

Outcomes	Number of events		Crude incidence rate		Model 1	Model 2	Model 3	Model 4
			per 1,000 person-years (95% CI)					
	No history of GDM	History of GDM	No history of GDM	History of GDM	HR (95% CI)	HR (95% CI)	HR (95% CI)	HR (95% CI)
Total cardiovascular outcome	12,984/203,249	110/1276	5.81 (5.71–5.91)	7.80 (6.34–9.26)	1.53 (1.34–1.76)	1.37 (1.19–1.56)	1.37 (1.19–1.56)	1.36 (1.18–1.55)
Coronary artery disease	5,675/213,822	57/1338	2.59 (2.52–2.65)	4.19 (3.10–5.28)	1.67 (1.38–2.01)	1.33 (1.10–1.61)	1.33 (1.10–1.61)	1.31 (1.08–1.59)
Myocardial infarction	2,108/215,909	30/1358	0.94 (0.90–0.98)	2.13 (1.37–2.89)	2.16 (1.66–2.80)	1.67 (1.29–2.18)	1.68 (1.29–2.19)	1.65 (1.27–2.15)
Ischemic stroke	1,268/217,478	17/1387	0.57 (0.54–0.60)	1.21 (0.63–1.78)	2.10 (1.48–2.98)	1.70 (1.19–2.42)	1.69 (1.19–2.41)	1.68 (1.18–2.39)
Peripheral artery disease	867/217,552	13/1385	0.39 (0.36–0.41)	0.92 (0.42–1.43)	2.47 (1.68–3.65)	1.73 (1.16–2.56)	1.73 (1.16–2.56)	1.69 (1.14–2.51)
Heart failure	2,222/217,322	26/1377	1.00 (0.95–1.04)	1.86 (1.14–2.57)	1.86 (1.40–2.45)	1.43 (1.08–1.89)	1.43 (1.08–1.90)	1.41 (1.06–1.87)
Aortic stenosis	641/217,757	3/1390	0.29 (0.26–0.31)	0.21 (0.03–0.45)	1.07 (0.48–2.39)	0.85 (0.38–1.91)	0.85 (0.38–1.90)	0.84 (0.37–1.87)
Mitral regurgitation	861/217,593	13/1,386	0.39 (0.36–0.41)	0.92 (0.42–1.43)	2.48 (1.68–3.65)	2.28 (1.53–3.38)	2.26 (1.52–3.36)	2.25 (1.51–3.34)
Atrial fibrillation/flutter	4,691/215,787	40/1383	2.12 (2.06–2.18)	2.85 (1.97–3.73)	1.62 (1.30–2.02)	1.48 (1.18–1.84)	1.48 (1.18–1.84)	1.47 (1.18–1.84)
Venous thromboembolism	3,217/209,543	20/1342	1.50 (1.44–1.55)	1.47 (0.82–2.11)	1.10 (0.80–1.50)	1.06 (0.78–1.46)	1.06 (0.78–1.46)	1.06 (0.77–1.45)

Data are presented as proportion (%)

*CI* confidence interval, *GDM* gestational diabetes mellitus

Model 1, adjusted for age, race, BMI, smoking by Cox proportional hazards regression analysis

Model 2, adjusted for age, race, BMI, smoking, prevalent diseases (hypertension, diabetes, or hypercholesterolemia) by Cox proportional hazards regression analysis

Model 3, adjusted for age, race, BMI, smoking, prevalent diseases (hypertension, diabetes, or hypercholesterolemia), and medication (aspirin, anti-hypertensive, and cholesterol-lowering agent) by Cox proportional hazards regression analysis

Model 4*,* adjusted for age, race, BMI, smoking, alcohol consumption, prevalent diseases (hypertension, diabetes, or hypercholesterolemia), medication (aspirin, Anti-hypertensive, and cholesterol-lowering agent), early menopause, and hysterectomy by Cox proportional hazards regression analysis

**Fig. 5:**
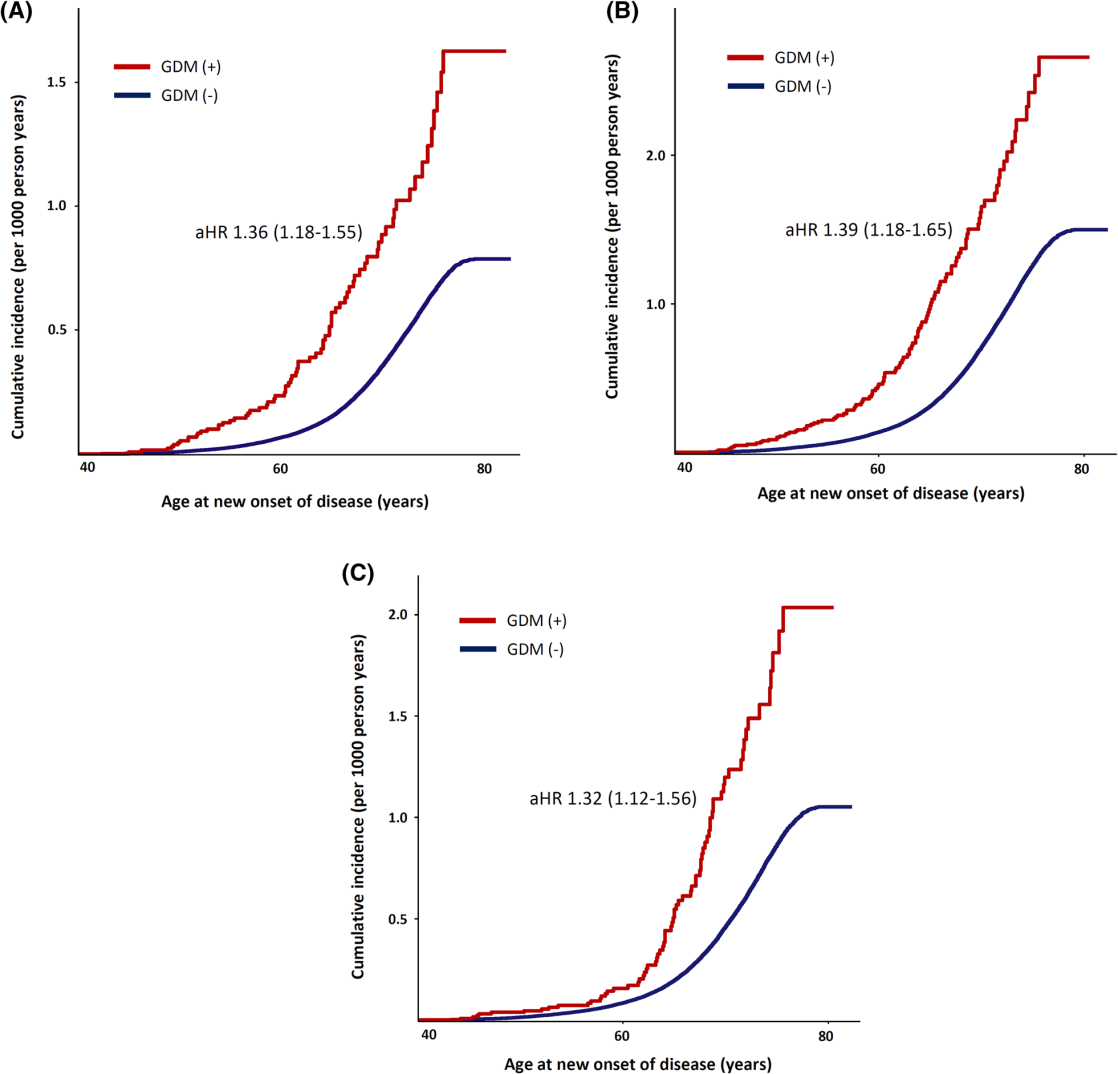
Survival analysis of total cardiovascular outcomes (**a**) Total cardiovascular outcome (**b**) Atherosclerotic cardiovascular disease ^§^ (**c**) Non-atherosclerotic cardiovascular disease. ^§^ a composite of coronary artery disease, myocardial infarction (MI), ischemic stroke, and peripheral artery disease a composite of heart failure, aortic stenosis, mitral regurgitation, atrial fibrillation/ flutter, and venous thromboembolism aHR: adjusted hazard ratio [adjusted for age, race, BMI, smoking, alcohol consumption, early menopause, hysterectomy, prevalent diseases (hypertension, diabetes, or dyslipidemia), and medication (aspirin, anti-hypertensive, and cholesterol-lowering agent) by Cox proportional hazards regression analysis]

Figure 5 compares the cumulative incidence of cardiovascular outcomes between women with a history of GDM and those without, plotted against participants’ age. GDM was associated with the long-term risk of cardiovascular disease. A composite of ASCVD (a composite of coronary artery disease, myocardial infarction, ischemic stroke, and peripheral artery disease) and a composite of non-ASCVD (a composite of heart failure, aortic stenosis, mitral regurgitation, atrial fibrillation/flutter, and venous thromboembolism) were also increased in women with a history of GDM. Additional file 1: Figure S1 shows the cumulative incidence of each cardiovascular outcome that was significantly different between the two groups of cases.

To reduce the bias due to covariates in the evaluation of the effect of GDM, further analysis after propensity score matching was also performed. As a result of matching, there were no significant differences in major clinical characteristics between 1,380 cases with a history of GDM and 6,900 control cases (Additional file 1: Table S3). After matching, cases with GDM had a higher risk for the new occurrence of total cardiovascular outcome, coronary artery disease, myocardial infarction, ischemic stroke, peripheral artery disease, heart failure, mitral regurgitation, and atrial fibrillation/flutter (Table 3).

**Table 3:** Incident diagnosis of cardiovascular outcomes in subgroup after propensity score matching

Outcomes	No history of GDM	History of GDM	HR^a^ (95% CI)
	(n = 6,900)	(n = 1,380)	
Total cardiovascular outcome	399 (6.3%)	108 (8.5%)	1.33 (1.14–1.55)
Coronary artery disease	211 (3.2%)	55 (4.1%)	1.30 (1.05–1.61)
Myocardial infarction	74 (1.1%)	29 (2.2%)	1.69 (1.25–2.30)
Ischemic stroke	50 (0.7%)	16 (1.2%)	1.46 (0.98–2.18)
Peripheral artery disease	25 (0.4%)	13 (0.9%)	2.08 (1.29–3.35)
Heart failure	87 (1.3%)	25 (1.8%)	1.42 (1.03–1.95)
Aortic stenosis	20 (0.3%)	3 (0.2%)	0.86 (0.36–2.02)
Mitral regurgitation	20 (0.3%)	13 (0.9%)	2.92 (1.59–5.38)
Atrial fibrillation/flutter	138 (2.0%)	40 (2.9%)	1.39 (1.09–1.79)
Venous thromboembolism	99 (1.5%)	20 (1.5%)	1.02 (0.73–1.44)

The control group was selected after matching for age, race, BMI, smoking history, alcohol consumption, early menopause, hysterectomy, prevalent morbidities (diabetes, hypertension, dyslipidemia), and medications (aspirin, anti-hypertensives, cholesterol-lowering agent). Data are presented as proportion (%)

^a^Adjusted for age, race, BMI, smoking, alcohol consumption, early menopause, hysterectomy, prevalent diseases (hypertension, diabetes, or dyslipidemia), and medication (aspirin, anti-hypertensive, and cholesterol-lowering agent) by Cox proportional hazards regression analysis

### Causal mediation analysis to evaluate the effect of prevalent comorbidities

In addition, we performed causal mediation analysis to examine the contribution of well-known risk factors to observed cardiovascular risk. Mediation analysis suggested that diabetes explained 23%, hypertension explained 11%, and dyslipidemia explained 10% of the association between GDM and overall cardiovascular outcome (Table 4). For both atherosclerotic cardiovascular disease and non-atherosclerotic cardiovascular disease, diabetes, hypertension, and dyslipidemia showed mediation effect, but the proportion of the mediated effect of chronic metabolic diseases was higher in atherosclerotic cardiovascular disease than non-atherosclerotic cardiovascular disease.

**Table 4:** Causal mediation analysis to evaluate the mediative effect of prevalent comorbidities on the association between gestational diabetes and long-term cardiovascular outcomes

	Proportion of mediated effect by diabetes	Proportion of mediated effect by hypertension	Proportion of mediated effect by dyslipidemia
Total cardiovascular outcome	0.21 (0.14–0.36)	0.11 (0.08–0.17)	0.10 (0.07–0.16)
ASCVD	0.30 (0.21–0.49)	0.12 (0.09–0.19)	0.14 (0.10–0.20)
Coronary artery disease	0.34 (0.23–0.63)	0.15 (0.10–0.25)	0.18 (0.13–0.31)
Myocardial infarction	0.26 (0.18–0.47)	0.09 (0.06–0.16)	0.10 (0.07–0.16)
Ischemic stroke	0.23 (0.14–0.55)	0.08 (0.05–0.19)	0.08 (0.05–0.21)
Peripheral artery disease	0.31 (0.20–0.75)	0.11 (0.08–0.30)	0.11 (0.07–0.24)
Non-ASCVD	0.19 (0.12–0.40)	0.11 (0.07–0.22)	0.08 (0.05–0.16)
Heart failure	0.35 (0.24–0.83)	0.12 (0.08–0.30)	0.12 (0.08–0.30)
Mitral regurgitation	0.06 (-0.04–0.18)	0.06 (0.04–0.12)	0.05 (0.02–0.12)
Atrial fibrillation/flutter	0.13 (0.07–0.32)	0.12 (0.08–0.26)	0.08 (0.04–0.16)

*ASCVD* atherosclerotic cardiovascular disease, *non-ASCVD* non-atherosclerotic cardiovascular disease

### The risk of long-term cardiovascular outcomes from the index pregnancy

Considering the time interval between the index pregnancy and enrollment, we further analyzed the cardiovascular outcome from the index pregnancy in 182,240 women whose age at the index pregnancy was available (n = 634 in women with GDM history and n = 181,606 in women without GDM history). The index pregnancy was defined as the pregnancy diagnosed as GDM or the first pregnancy in women without GDM history. Among these, women with history of CVD before pregnancy (n = 577) were excluded, remaining 181,663 women in the analysis. As a result, the risk of both ASCVD and non-ASCVD from the index pregnancy was increased in women with a history of GDM than in those without (Additional file 1: Figure S2). Specifically, the risk of the total cardiovascular outcome, coronary artery disease, myocardial infarction, ischemic stroke, peripheral artery disease, heart failure, mitral regurgitation, and atrial fibrillation/flutter were increased in women with a history of GDM, even after adjustment for covariates by Cox proportional hazards regression analysis (Table 5).

**Table 5:** Incident diagnosis of cardiovascular outcomes from the index pregnancy

Outcomes	HR (95% CI)^a^
Total cardiovascular outcome	2.06 (1.72–2.47)
Coronary artery disease	3.31 (2.55–4.31)
Myocardial infarction	2.19 (1.30–3.70)
Ischemic stroke	3.30 (1.77–6.15)
Peripheral artery disease	5.13 (2.87–9.17)
Heart failure	2.43 (1.30–4.53)
Aortic stenosis	1.82 (0.45–7.40)
Mitral regurgitation	2.89 (1.29–6.49)
Atrial fibrillation/ flutter	1.94 (1.25–3.02)
Venous thromboembolism	1.24 (0.88–1.75)

^**a**^adjusted for age at delivery, race, and prevalent diseases before pregnancy (hypertension, diabetes, or hypercholesterolemia) by Cox proportional hazards regression analysis

### The risk of each cardiovascular disease in women without any prevalent cardiovascular disease

To reduce potential effect from prevalent CVD on the development of incident each CVD, we performed a sensitivity analysis in women without any prevalent CVD at enrollment. As a result, GDM history increased the risk of various cardiovascular outcomes even in women without prevalent CVD, including total cardiovascular outcome, coronary artery disease, myocardial infarction, ischemic stroke, peripheral artery disease, mitral regurgitation, and atrial fibrillation/flutter (Additional file 1: Table S4).

In addition, we also analyzed the risk of a new occurrence of each cardiovascular outcome without any prior cardiovascular disease. For that analysis, the occurrence of cardiovascular outcome in the presence of any prior cardiovascular disease was excluded. For example, if the patient developed coronary artery disease and then new heart failure subsequently during follow-up, the patient was excluded from the analysis of heart failure risk. As a result, women with a history of GDM had an increased risk of various cardiovascular outcomes, including coronary artery disease, myocardial infarction, peripheral artery disease, mitral regurgitation, and atrial fibrillation/flutter, in the absence of any prior cardiovascular disease (Additional file 1: Table S5).

## Discussion

In the current study, women with a history of GDM had a greater risk for diabetes and had an increased risk for total cardiovascular outcomes. Specifically, women with a history of GDM had an increased risk for the new occurrence of coronary artery disease, myocardial infarction, ischemic stroke, peripheral artery disease, heart failure, mitral regurgitation, and atrial fibrillation/flutter.

Physiologic changes during pregnancy, such as circulatory volume increases, inflammatory changes, insulin resistance and hyperlipidemia, can be cardiovascular and metabolic challenges in pregnant women [28]. Because of these changes, pregnancy complications such as gestational diabetes, preeclampsia, preterm delivery, and small/large fore gestational age can develop, and it has been reported that women who experience pregnancy complications are likely to develop the further cardiovascular disease after pregnancy [3–8]. Therefore, pregnancy can be a chance of a period to identify women at high risk for long-term cardiovascular disease [29]. Clinical guidelines recommend considering pregnancy complications as a possible risk factor for cardiovascular disease [30–34].

For GDM, there have been several studies regarding the increased risk for subsequent atherosclerotic cardiovascular disease [4–6, 17–23]. Among these studies, few studies followed up women 20–30 years after pregnancy. In the Nurses' Health Study II, participants with a mean age of 35 years at enrollment were followed up for a median of 25.7 years and the study showed that cardiovascular disease, which was defined as self-reported myocardial infarction or stroke, was higher in women with a history of GDM [20]. In a retrospective cohort study from Canada, women with GDM were followed up for a maximum of 25 years after delivery, and women with GDM had a higher history of ischemic heart disease, myocardial infarction, and coronary angioplasty [22]. In a case–control study from the United Kingdom, women were followed up for a median of 2.9 years (a maximum of 25 years), and women with GDM had an increased risk for ischemic heart disease [23]. In the current study, we also showed that women with a history of GDM were at increased risk for long-term cardiovascular outcomes. The current study has several strengths compared to previous studies. First, as the UK Biobank enrolled participants at 40–69 years old, we have a chance to look at the long-term outcome after pregnancy. In the current study population, the women were enrolled at the mean age of 56.9 years old, with a mean duration between first birth and enrollment of 32 years, and were followed up for a mean of 10.3 years. Because of this characteristic of the study population, we could compare the incidence of cardiovascular outcomes in an elderly population up to a mean of 42 years after the first delivery. Although previous studies have shown an increased risk for cardiovascular disease closer to the time of pregnancy after GDM, these young women are at still low absolute risk for CVD. In the latest guideline, one of the recommended targets for statin therapy is adults aged 40–75 years at high risk. Despite the controversy, middle aged women at high risk for CVD are still candidates for aspirin prophylaxis for primary prevention of CVD [35]. Therefore, the result of the current study evokes the next question, whether women with GDM history may benefit from cardiovascular preventive strategies, such as intensive lifestyle modification, pharmacologic treatment including statin or aspirin therapy in midlife. Second, as the UK Biobank has been collecting data from various sources after enrollment, we could also capture various cardiovascular outcomes after enrollment. As a result, we found that GDM was associated with not only atherosclerotic cardiovascular outcomes but also non-atherosclerotic cardiovascular outcomes.

In the current study, we showed that women with prior GDM are at increased risk for not only atherosclerotic cardiovascular disease but also other diverse cardiovascular diseases such as heart failure, mitral regurgitation, and atrial fibrillation/flutter. According to our results, the increased risk of non-atherosclerotic cardiovascular disease in women with prior GDM history could also be partly explained by chronic metabolic comorbidities, although the mediation effect is less than the atherosclerotic cardiovascular disease outcome. In addition, the associations of overt DM with non-atherosclerotic cardiovascular disease have been previously reported, although there is a lack of knowledge regarding the association between GDM and non-atherosclerotic cardiovascular disease.

Regarding this relationship, several mechanisms may be considered. Dysglycemia and metabolic syndrome are associated with myocardial dysmetabolism, myocardial fibrosis, atrial or ventricular remodeling, and pericardial fat accumulation which can be possible drivers for the association between GDM and non-ASCVD [36]. For atrial fibrillation/flutter, the underlying pathophysiology of increased risk in patients with diabetes is attributed to structural, electrical/electromechanical, and autonomic changes [37, 38]. Mitral regurgitation can be linked to diabetes in terms of ischemic mitral regurgitation after coronary artery disease [39]. However, several anecdotal reports have suggested other causes of mitral regurgitation such as autonomic dysregulation, which is the known consequence of diabetes, or endocrine autoimmune disease [40, 41]. The most common valvular heart disease in patients with diabetes is functional mitral regurgitation, and diabetic cardiomyopathy is associated with LV remodeling and causes mitral annular dilation, papillary muscle displaces, and mitral valve insufficiency [42]. Heart failure is also known to be increased in diabetes patients, and there is an increased recognition that this relationship persists independent of coronary artery disease [43]. The Swedish AMORIS cohort study showed that impaired fasting glucose was associated with the development of AF and heart failure during the follow-up periods of 19.1 years, even before it reaches levels for overt diabetes [44]. Further studies are needed to determine whether these pathophysiologic mechanisms might also be applicable to the relationship between GDM and diverse cardiovascular outcomes.

The current study has several limitations. First, the UK Biobank collected baseline characteristics both from participants’ responses to the questionnaire (self-report) and from nationwide hospital or death registry data, and there can be recall bias in self-report data for GDM. Second, the prevalence of GDM in the study population was less than 1%, quite less than the reported prevalence of GDM in modern obstetrics. However, universal screening for GDM has been a routine practice in obstetrics since the late 1990s [45], and before that period, GDM was reported to have a prevalence of 1–2% [46] and different screening and diagnostic criteria became proposed, although antenatal screening for GDM had been gradually established [47]. Given the time period between first live birth and enrollment in this study [mean duration 32 years, between first live birth and enrollment (2006–2010)], the majority of women in the UK Biobank are most likely to have been pregnant earlier than this universal GDM screening period (late 1990s). Therefore, many cases of GDM may have gone undiagnosed and been included in the control group of the current study, resulting in an underestimation of the effect size of GDM on subsequent cardiovascular outcomes. Thus, further studies are needed to determine the long-term effect of GDM after the period when GDM has been routinely screened for all pregnant women. In addition, we could not capture the number of GDM occurrences among several pregnancies in one woman, we could not evaluate the impact of recurrent GDM on long-term cardiovascular outcomes. Third, concerns in the UK Biobank on ‘healthy volunteer’ selection bias may also play a role in the relatively lower prevalence of GDM. The UK Biobank participants are less likely to be obese, to smoke, and drink, and more likely to live in areas with higher socioeconomic status [48]. Therefore, we need to consider the healthy volunteer effect when studying this population, which is consistent with other prospective cohorts. Although UK Biobank may not be the best cohort for generalizable estimation of prevalence or incidence of disease, investments into exposure-disease association studies using the UK Biobank may be generalizable with a sufficient number of participants [48, 49], but further studies with a nationwide cohort will be needed to validate our findings. Fourth, as our definitions of comorbidity and cardiovascular outcomes were based on ICD codes, the misclassification of diagnosis is possible. In addition, the limited number of some CVD outcomes in the group with GDM, including ischemic stroke, heart failure, mitral regurgitation, and atrial fibrillation/flutter, weakened the statistical power of the results. Because of the small number of outcomes, we could not adjust for all the possible covariates that may influence cardiovascular disease. In addition, the primary outcome of the current study was CVD risk after enrollment, not after the index pregnancy. A more appropriate analytic method would be the evaluation of incident cardiovascular outcome after index pregnancy while adjusting multiple co-variates at the time of index pregnancy. However, because of the limitation of UK biobank which enrolled participants at 40–69 years old, retrieved various information at enrollment, and followed up after enrollment, we mainly analyzed the risk of CVD from enrollment, to adjust for various lifestyle covariates [BMI, smoking, alcohol consumption, medication (aspirin, anti-hypertensive, and cholesterol-lowering agent), early menopause, and hysterectomy] which were not available at the time of index pregnancy. In addition, by analyzing the CVD risk after enrollment, we could also analyze the mediation effect of co-morbidities such as diabetes, hypertension, dyslipidemia in the development of CVD. Further prospective studies are needed to evaluate the risk of CVD after index pregnancy, adjusting for various covariates. Last, further studies are needed from non-European regions, because the current study population only includes women in the United Kingdom.

## Conclusion

In conclusion, we found that women with a GDM history are at increased risk of long-term cardiovascular complications. Moreover, GDM is associated with an increased risk of diverse cardiovascular outcomes. Chronic metabolic comorbidities, such as diabetes, hypertension, and dyslipidemia, partly explain the onset of cardiovascular outcomes in women with a GDM history. Our findings suggest that regular screening and long-term proper strategies for CVD prevention are needed for women with a history of GDM.

## Supplementary Information

**Additional file 1: Table S1.** ICD-9 and ICD-10 codes for disease definition for gestational diabetes, congenital heart disease (for exclusion) and cardiovascular outcomes. **Table S2.** Frequency of prevalent cardiovascular diseases at enrolment. **Table S3.** Baseline clinical features and prevalent diseases after propensity score matching. Control group was selected after matching for age, race, BMI, smoking history, alcohol consumption, prevalent morbidities (diabetes, hypertension, dyslipidemia), and medications (aspirin, anti-hypertensives, cholesterol lowering agent). **Table S4.** The risk incident cardiovascular outcomes in women without prevalent cardiovascular disease. **Table S5.** The risk incident cardiovascular outcomes in women without any prior cardiovascular disease. **Figure S1.** Survival analysis of each cardiovascular outcome. **Figure S2.** Survival analysis of each cardiovascular outcome from the index pregnancy.

## Data Availability

All researchers in academic, commercial and charitable settings can apply to use the UK Biobank resource for health-related research in the public interest (www.ukbiobank.ac.uk/registerapply/).

## Abbreviations

ASCVD: Atherosclerotic cardiovascular disease
BMI: Body mass index
CVD: Cardiovascular disease
GDM: Gestational diabetes mellitus
HR: Hazard ratio
ICD: International classification of disease
