# Supplementary material for: Long-term cardiovascular outcomes of gestational diabetes mellitus: a prospective UK Biobank study
Source: Cardiovasc Diabetol. 2022 Oct 29;21:221. doi: 10.1186/s12933-022-01663-w (PMC9618212; doi:10.1186/s12933-022-01663-w)
Supplement: Supplementary file 1 — Additional file 1: Table S1. ICD-9 and ICD-10 codes for disease definition for gestational diabetes, congenital heart disease (for exclusion) and cardiovascular outcomes. Table S2. Frequency of prevalent cardiovascular diseases at enrolment. Table S3. Baseline clinical features and prevalent diseases after propensity score matching. Control group was selected after matching for age, race, BMI, smoking history, alcohol consumption, prevalent morbidities (diabetes, hypertension, dyslipidemia), and medications (aspirin, anti-hypertensives, cholesterol lowering agent). Table S4. The risk incident cardiovascular outcomes in women without prevalent cardiovascular disease. Table S5. The risk incident cardiovascular outcomes in women without any prior cardiovascular disease. Figure S1. Survival analysis of each cardiovascular outcome. Figure S2. Survival analysis of each cardiovascular outcome from the index pregnancy. [file 12933_2022_1663_MOESM1_ESM.docx]

**Additional file Table S1.** ICD-9 and ICD-10 codes for disease definition for gestational diabetes, congenital heart disease (for exclusion) and cardiovascular outcomes

| **Disease** | **ICD codes** |
| --- | --- |
| Gestational diabetes | ICD 9: 6480, 6488  ICD 10: O244 |
| Congenital heart disease | ICD 9: 745, 7450, 74500, 74501, 7451, 74510, 74511, 74512, 74518, 74519, 7452, 74520, 74521, 7453, 7454, 74540, 74541, 74542, 74548, 74549, 7455, 74550, 74551, 74552, 74558, 74559, 7456, 74560, 74561, 74562, 74563, 74568, 74569, 7457, 7458, 7459, 746, 7460, 74600, 74601, 74602, 74608, 74609, 7461, 7462, 7463, 7464, 7465, 7466, 7467, 7468, 74680, 74681, 74682, 74683, 74684, 74685, 74686, 74687, 74688, 7469, 74690, 74691, 74692, 74693, 74699, 747, 7470, 7471, 74710, 74711, 74719, 7472, 74720, 74721, 74722, 74723, 74724, 74725, 74726, 74727, 74728, 74729, 7473, 74730, 74731, 74732, 74733, 74734, 74738, 74739, 7474, 74740, 74741, 74742, 74743, 74744, 74745, 74748, 74749  ICD 10: Q20, Q200, Q201, Q202, Q203, Q204, Q205, Q206, Q208, Q209, Q21, Q210, Q211, Q212, Q213, Q214, Q218, Q219, Q22, Q220, Q221, Q222, Q223, Q224, Q225, Q226, Q228, Q229, Q23, Q230, Q231, Q232, Q233, Q234, Q238, Q239, Q24, Q240, Q241, Q242, Q243, Q244, Q245, Q246, Q248, Q249, Q25, Q250, Q251, Q252, Q253, Q254, Q255, Q256, Q257, Q258, Q259, Q26, Q260, Q261, Q262, Q263, Q264, Q265, Q266, Q268, Q269 |
| Hypertension | ICD 9: 401, 4010, 4011, 4019, 402, 4020, 4021, 4029, 403, 4030, 4031, 4039, 404, 4040, 4041, 4049, 405, 4050, 4051, 4059  ICD 10: I10, I11, I110, I119, I12, I120, I129, I13, I130, I131, I132, I139, I15, I150, I151, I152, I158, I159 |
| Dyslipidemia | ICD 10: E780, E781, E782, E784, E785 |
| Diabetes mellitus | ICD 9: 250, 2500, 25000, 25001, 25009, 2501, 25010, 25011, 25019, 2502, 25020, 25021, 25029, 2503, 2504, 2505, 2506, 2507, 2509, 25090, 25091, 25099  ICD 10: E10, E100, E101, E102, E103, E104, E105, E106, E107, E108, E109, E11, E110, E111, E112, E113, E114, E115, E116, E117, E118, E119, E14, E140, E141, E142, E143, E144, E145, E146, E147, E148, E149 |
| Coronary artery disease | ICD 9: 410, 4109, 411, 4119, 412, 4129, 4140, 4148, 4149  ICD 10: I21, I210, I211, I212, I213, I214, I219, I22, I220, I221, I228, I229, I23, I230, I231, I232, I233, I234, I235, I236, I238, I24, I240, I241, I248, I249, I251, I252, I255, I256, I258, I259 |
| Peripheral arterial disease | ICD 9: 4400, 4402, 4438, 4439  ICD 10: I70, I700, I7000, I7001, I702, I7020, I7021, I708, I7080, I709, I7090, I738, I739 |
| Heart failure | ICD 9: 4254, 4280, 4281, 4289  ICD 10: I110, I130, I132, I255, I420, I421, I422, I425, I428, I429, I50, I500, I501, I509 |
| Aortic stenosis | ICD 10: I060, I062, I350, I352 |
| Mitral regurgitation | ICD 9: 3942  ICD 10: I051, I052, I340 |
| Atrial fibrillation or flutter | ICD 9: 4273  ICD 10: I48, I480, I481, I482, I483, I484, I489 |
| Venous thromboembolism | ICD 9: 4151, 4511  ICD 10: I26, I260, I269, I80, I800, I801, I802, I803, I808, I809, I81, I820, I822, D68, D680, D681, D682, D683, D684, D685, D686, D688, D689 |

**Additional file Table S2.** Frequency of prevalent cardiovascular diseases at enrollment

| **Outcomes** | **No history of GDM** | **History of GDM** |
| --- | --- | --- |
|  | **(n = 217,940)** | **(n = 1,390)** |
| Total cardiovascular outcome | 14,691 (6.7) | 114 (8.2) |
| Coronary artery disease | 4,118 (1.9) | 52 (3.7) |
| Myocardial infarction | 2,031 (0.9) | 32 (2.3) |
| Ischemic stroke | 462 (0.2) | 3 (0.2) |
| Peripheral artery disease | 388 (0.2) | 5 (0.4) |
| Heart failure | 618 (0.3) | 13 (0.9) |
| Aortic stenosis | 183 (0.1) | 0 (0.0) |
| Mitral regurgitation | 347 (0.2) | 4 (0.3) |
| Atrial fibrillation/ flutter | 2153 (1.0) | 7 (0.5) |
| Venous thromboembolism | 8,397 (3.9) | 48 (3.5) |

Data are presented as proportion (%).

Abbreviations: GDM, gestational diabetes mellitus

**Additional file Table S3.** Baseline clinical features and prevalent diseases after propensity score matching. Control group was selected after matching for age, race, BMI, smoking history, alcohol consumption, prevalent morbidities (diabetes, hypertension, dyslipidemia), and medications (aspirin, anti-hypertensives, cholesterol lowering agent).

| **Characteristics** | **No history of GDM** | **History of GDM** |
| --- | --- | --- |
|  | **(n = 6,900)** | **(n = 1,380)** |
| Age (years) | 52.2 ± 8.1 | 52.1 ± 8.1 |
| Caucasian | 5,761 (83.5) | 1,146 (83.0) |
| BMI (kg/m^2^) | 29.2 ± 6.6 | 29.2 ± 6.0 |
| Obesity (BMI >30 kg/m^2^) | 2,580 (37.4) | 521 (37.8) |
| Early menopause <40 years | 221 (3.2) | 46 (3.3) |
| Hysterectomy | 440 (6.4) | 91 (6.6) |
| Ever smoking | 2,561 (37.1) | 498 (36.1) |
| Prevalent comorbidity at baseline |  |  |
| Type 2 diabetes | 2,076 (30.1) | 411 (29.8) |
| Hypertension | 2,484 (36.0) | 497 (36.0) |
| Dyslipidemia | 1,455 (21.1) | 300 (21.7) |
| Use of medication |  |  |
| Aspirin | 1,191 (17.3) | 232 (16.8) |
| Anti-hypertensive agent | 1,916 (27.8) | 384 (27.8) |
| Lipid-lowering agent | 1,942 (28.1) | 392 (28.4) |

Data are presented as proportion (%) or mean ± standard deviation.

Abbreviations: BMI, body mass index; GDM, gestational diabetes mellitus

**Additional file Table S4.** The risk incident cardiovascular outcomes in women without prevalent cardiovascular disease

| **Outcomes** | **Hazard ratio^§^** |
| --- | --- |
| Total cardiovascular outcome | 1.36 (1.18-1.55)*** |
| Coronary artery disease | 1.36 (1.11-1.66)** |
| Myocardial infarction | 1.58 (1.15-2.17)** |
| Ischemic stroke | 1.72 (1.14-2.59)** |
| Peripheral artery disease | 1.91 (1.22-3.00)** |
| Heart failure | 1.15 (0.77-1.73) |
| Aortic stenosis | 0.81 (0.30-2.17) |
| Mitral regurgitation | 2.23 (1.39-3.59)*** |
| Atrial fibrillation/ flutter | 1.38 (1.06-1.80)* |
| Venous thromboembolism | 1.05 (0.75-1.46) |

Significant codes: ‘***’ <0.001; ‘**’ <0.01 ;‘*’ < 0.05

**^§^** Adjusted for age, race, BMI, smoking, alcohol consumption, early menopause, hysterectomy, prevalent diseases (hypertension, diabetes, or hypercholesterolemia), and medication (aspirin, Anti-hypertensive, and cholesterol-lowering agent) by cox proportional hazards regression analysis

**Additional file Table S5.** The risk incident cardiovascular outcomes in women without any prior cardiovascular disease

| **Outcomes** | **Hazard ratio^§^** |
| --- | --- |
| Coronary artery disease | 3.29 (2.53-4.29)*** |
| Myocardial infarction | 2.17 (1.28-3.67)** |
| Ischemic stroke | 1.48 (0.90-2.43) |
| Peripheral artery disease | 1.96 (1.18-3.24)** |
| Heart failure | 0.96 (0.57-1.63) |
| Aortic stenosis | 0.98 (0.37-2.65) |
| Mitral regurgitation | 2.33 (1.36-3.99)** |
| Atrial fibrillation/ flutter | 1.44 (1.09-1.91)* |
| Venous thromboembolism | 1.01 (0.71-1.43) |

Significant codes: ‘***’ <0.001; ‘**’ <0.01 ;‘*’ < 0.05

**^§^** Adjusted for age, race, BMI, smoking, alcohol consumption, early menopause, hysterectomy, prevalent diseases (hypertension, diabetes, or hypercholesterolemia), and medication (aspirin, Anti-hypertensive, and cholesterol-lowering agent) by cox proportional hazards regression analysis

**Additional file Figure S1.** **Survival analysis of each cardiovascular outcome**

**(A) Coronary artery disease (B) Myocardial infarction**

**
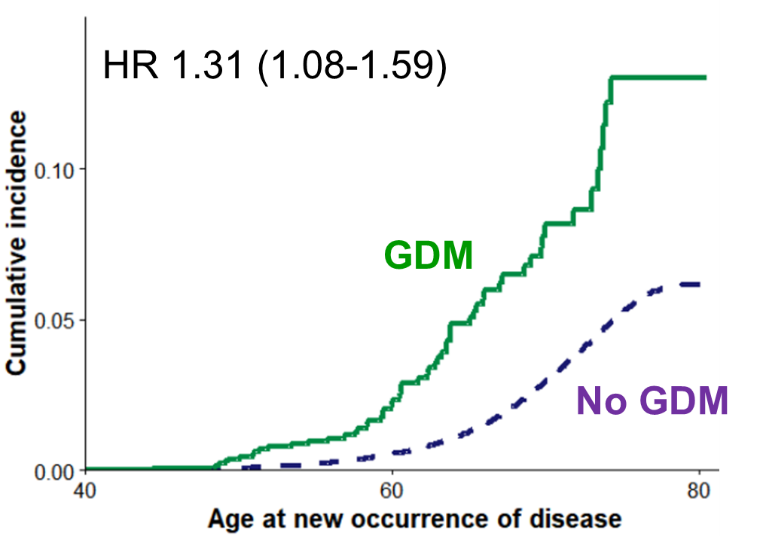

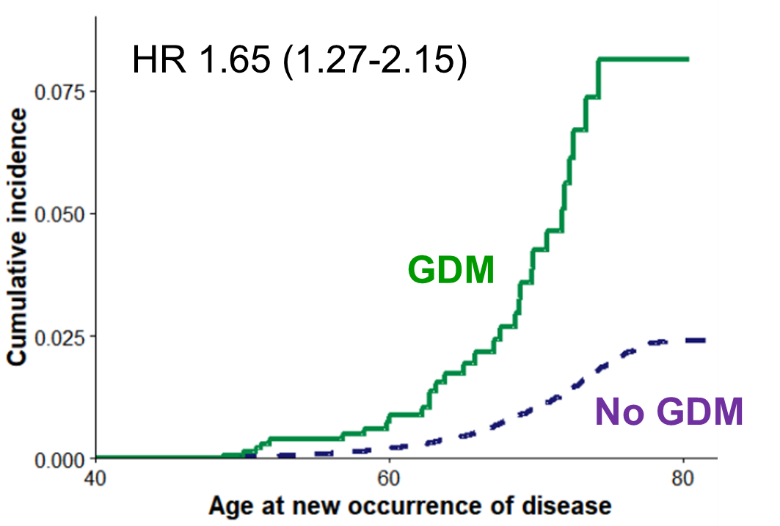
**

**(C) Ischemic stroke (D) Peripheral artery disease**

**
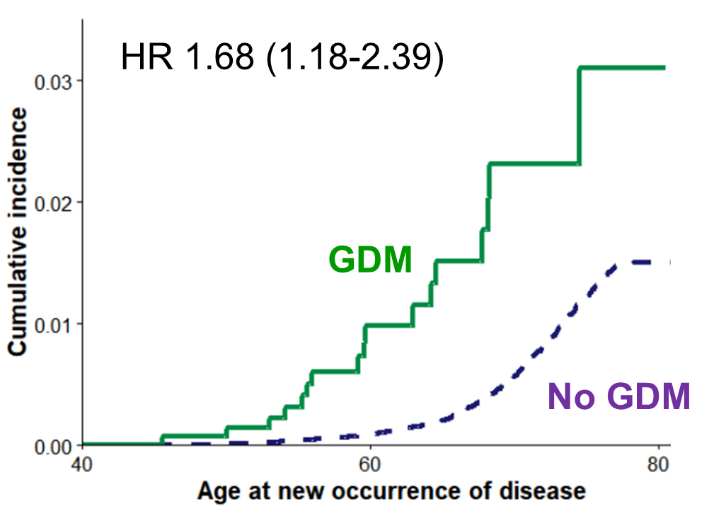

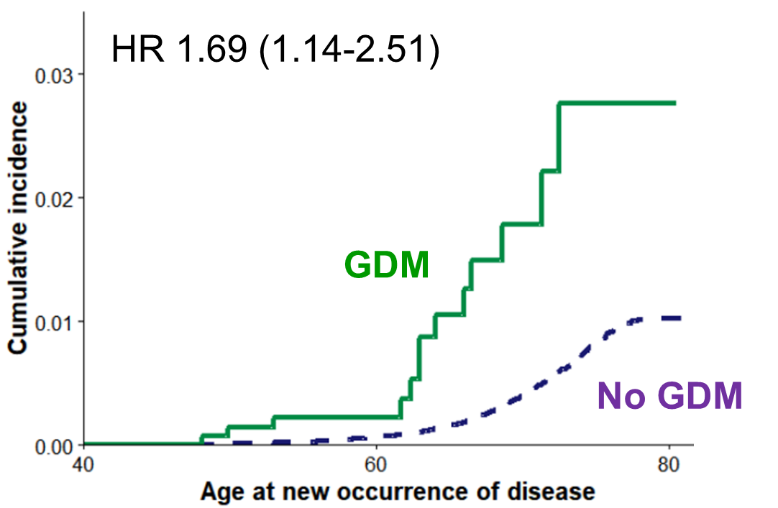
**

**(E) Heart failure (F) Mitral regurgitation**

**
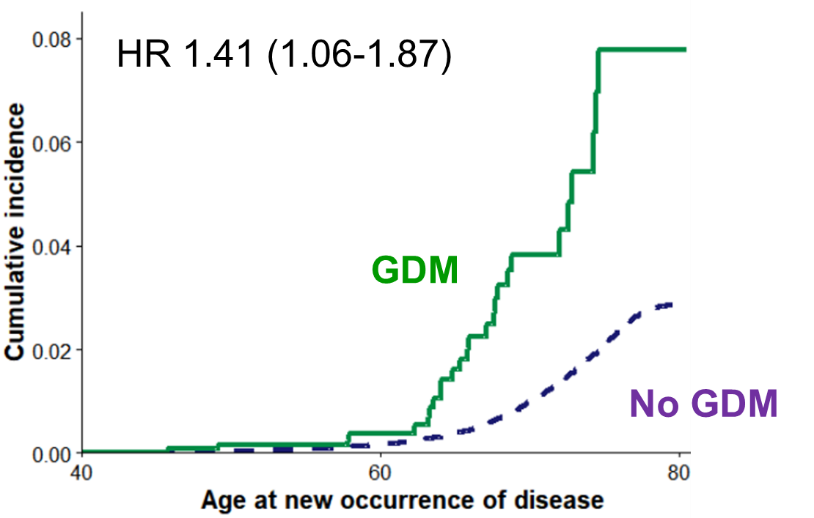

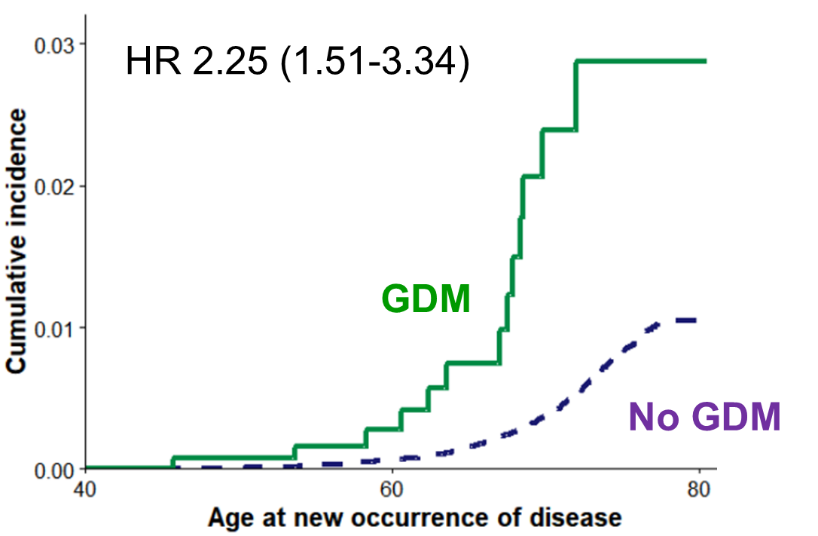
**

**(G) Atrial fibrillation/ flutter**

**
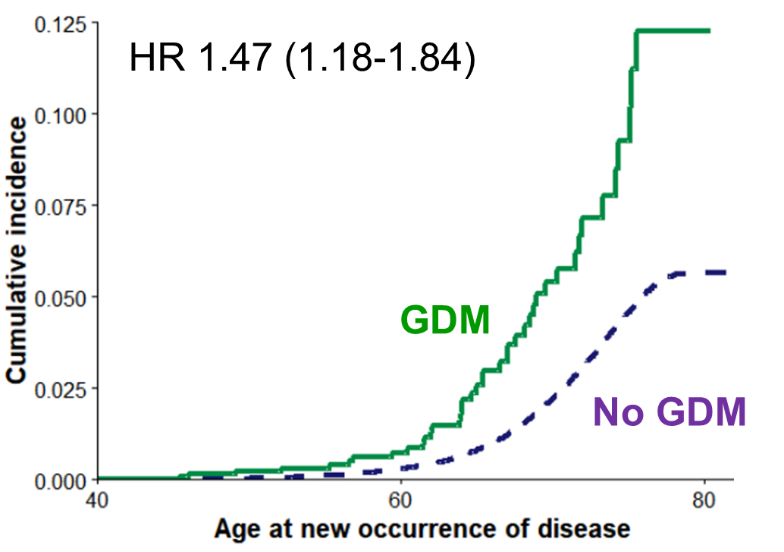
**

* adjusted for age, race, BMI, smoking, alcohol consumption, early menopause, hysterectomy, prevalent diseases (hypertension, diabetes, or hypercholesterolemia), and medication (aspirin, Anti-hypertensive, and cholesterol-lowering agent) by cox proportional hazards regression analysis

**Additional file Figure S2.** **Survival analysis of each cardiovascular outcome from the index pregnancy**

**(a) Atherosclerotic cardiovascular disease** ^§^

**
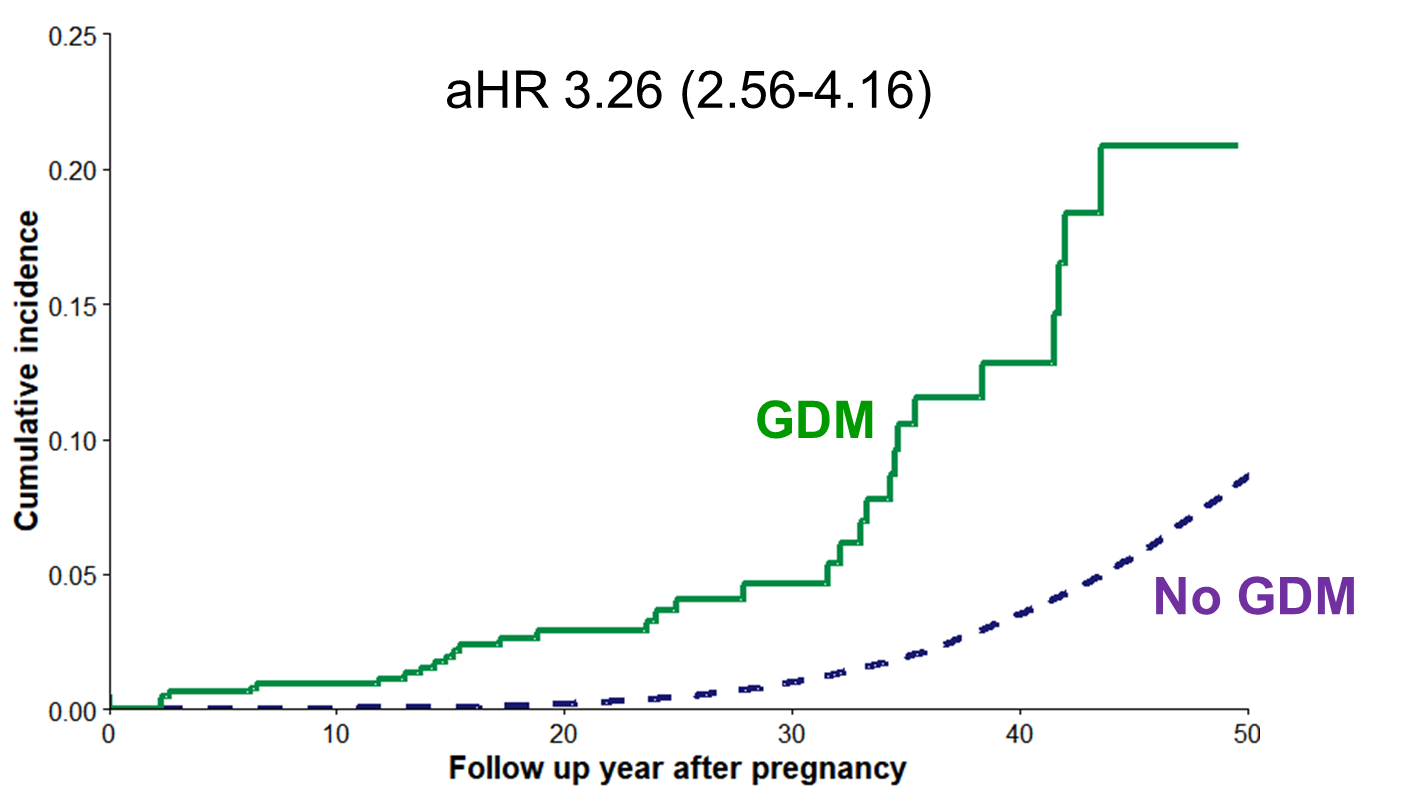
**

**(b) Non-atherosclerotic cardiovascular disease** ^¶^

**
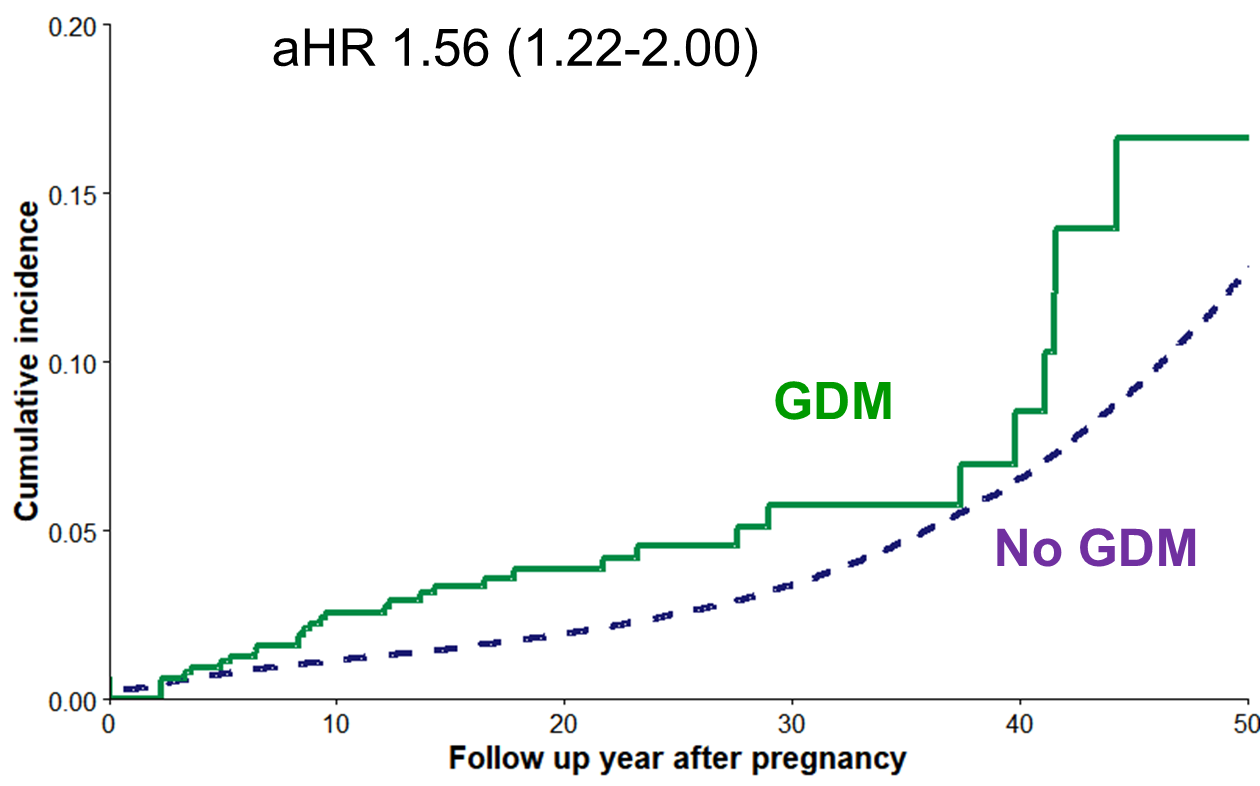
**

^§^ a composite of coronary artery disease, myocardial infarction (MI), ischemic stroke, and peripheral artery disease

^¶^ a composite of heart failure, aortic stenosis, mitral regurgitation, atrial fibrillation/ flutter, and venous thromboembolism

aHR: adjusted hazard ratio [adjusted for age at delivery, race, and prevalent diseases before pregnancy (hypertension, diabetes, or hypercholesterolemia) by Cox proportional hazards regression analysis]
